# The contagiousness of memes: containing the spread of COVID-19 conspiracy theories in a forensic psychiatric hospital

**DOI:** 10.1192/bjb.2020.120

**Published:** 2020-11-13

**Authors:** Reena Panchal, Alexander Jack

**Affiliations:** 1 Department of Psychiatry, Reaside Clinic, Birmingham and Solihull Mental Health Foundation Trust, UK; 2 Department of Psychiatry, Reaside Clinic, Birmingham and Solihull Mental Health Foundation Trust, UK

**Keywords:** COVID-19, forensic mental health services, conspiracy theory, meme, multidisciplinary working

## Abstract

COVID-19 has transformed healthcare service provision. In addition to the spread of a virus, there has been an equally concerning emergence and spread of conspiracy theories. Such theories can threaten societal cohesion and adherence to the necessary public health guidance. In a forensic in-patient setting, such difficulties can be amplified. In this paper, we outline the key theory in relation to the development and spread of conspiracy theory memes. We propose primary, secondary and tertiary level responses to tackle the possible generation and spread of harmful conspiracies in the forensic in-patient setting. We consider this to be important, as there is a risk that such beliefs could affect patients’ mental health and, in extremis, undermine physical health efforts to reduce the spread of COVID-19.

Forty-four cases of pneumonia of unknown microbial origin were reported to the World Health Organization (WHO) on 31 December 2019.^1^ Investigations revealed that the culprit organism was a novel coronavirus, dubbed COVID-19. COVID-19 has spread quicker than experts anticipated; the WHO declared an international state of emergency – a true pandemic – in early March 2020, as the virus spread rapidly between continents. The human cost has been, and continues to be, vast.

## The public health response

The global response to COVID-19 has emphasised the necessity for reduced close contact; hence, the intervention termed ‘social distancing’. To achieve this aim, many governments implemented ‘lockdown’ strategies to limit the free movement of the public, although the precise restrictions and severity of the measures have differed from country to country. The UK government urged people to ‘Stay Home, Save Lives, Protect the NHS’, with only essential travel permitted, restricted mixing of households and citizens at one point limited to a single exercise outing per day. There was a national drive to ‘flatten the curve’, with the stated intention to avoid overwhelming the National Health Service (NHS). A further patriotic message resonated with the public; that is, to protect the most vulnerable in society. Ultimately, the effectiveness of government and society's efforts to maintain this unconventional and rather antisocial injunction will be measured by the number of casualties.

## A forensic mental health hospital facing the pandemic

We are based in a psychiatric medium secure unit (MSU) in the West Midlands, UK. The MSU has capacity for 90 male patients across multiple wards and provides care to men who present with complex risk behaviours and experience psychopathology that warrants treatment under the Mental Health Act 1983 (amended 2007). The reality of COVID-19 within the MSU community parallels the changes seen in wider society. Initially, the virus was an abstract threat. However, measures were quickly implemented to increase hand-washing, social distancing and isolation of symptomatic patients.

At the time of writing, there have been 24 COVID-19 cases confirmed by positive swab in our MSU. Many patients experienced mild to moderate symptoms. However, three individuals required transfer to an intensive care unit for intubation and ventilation. Fortunately, they made a good recovery and returned to the MSU. Those with milder symptoms have been encouraged to self-isolate in their bedrooms. In anticipation of an increase in infections, one ward was designated to be the ‘COVID ward’, to contain the infection within a specific area and minimise further spread.

Clinical practice has changed significantly and it is hoped that, by mimicking national restrictions, the spread of COVID-19 will be contained. However, it is acknowledged that true ‘lockdown’ cannot be instated owing to the necessary travel between wards to maintain essential care.

## The Trojan horse: a split between staff and patients?

Patients in the MSU have been required to adjust to novel protocols (use of personal protective equipment (PPE), social distancing, etc.) while also processing the increased limitations on their movements at all times. Such dramatic changes, in the context of access to 24 h news coverage, have understandably heightened anxiety, fear and uncertainty. Of course, this is in addition to the usual physical and relational security provisions, such as air locks, keys and high fences that can be a source of angst for patients.

In these unprecedented conditions, we consider that there is a risk of an ‘us versus them’ dynamic developing, particularly as situational threat is high.^2^ On closed wards, it is plausible that staff are the vehicles of virus transmission, transporting the contagion onto the ward asymptomatically, given the incubation period of COVID-19. It is possible that some patients may view the movement of staff as contamination. The usual psychological containment that high staffing levels can provide may now be perceived as hostile, dangerous and unpredictable – when the danger cannot be seen, who is infected and imposing threat to the community? As the staff group fulfil the role of caregivers, a complicated role reversal ensues, as the caregiver is viewed as the source of danger: the Trojan horse entering the fortress.

## The emergence of conspiracy theories

We have noticed that conspiracy theories have emerged in tandem with the COVID-19 spread. Conspiracy theories have been defined as ‘attempts to explain the ultimate causes of significant social and political events and circumstances with claims of secret plots by two or more powerful actors’.^3^ Notably, conspiracy theories are similar to, though distinct from, misinformation and/or ‘conspiracy hypotheses’. Misinformation – false or inaccurate information, often intended to deceive (e.g. ‘fake news’) – can underpin conspiracy theories as it undermines mainstream narratives; conspiracy hypotheses are legitimate counter-narratives that can occur when there is uncertainty about the official story. Again, these can increase uncertainty and trust in a centralised message. The relationship between these phenomena is likely to be fluid.

Research has shown that conspiracy beliefs are common. For example, 60% of Americans believe that the CIA killed JFK^4^ and 46% of leave voters in the EU referendum believed that the vote would be rigged.^5^ There are many other types of theory that gather large follower groups.^3^ COVID-19 conspiracy theories have included fear of 5G broadband networks and persistent notions that the virus is man-made.^6^

Interestingly, individuals who hold one conspiracy theory are more likely to believe others,^7^ thus suggesting a possible underlying tendency to seek counter-narrative explanations and prefer them to information presented by institutions. Individuals who hold conspiracy beliefs are predominantly male, unmarried and of lower socioeconomic status. They are more likely to have weak social networks and belong to ethnic minority groups. Notably, they are likely to have had adverse childhoods and experience psychiatric problems as adults.^8^ Such demographics are highly consistent with a typical in-patient forensic population.^9,10^

Particular environmental conditions and psychological processes have been mooted to underpin such beliefs. In a review, Douglas et al^11^ identified three psychological motivations that led to a preference for conspiracy explanations: epistemic, existential and social. Each has particular relevance to the patient group in an MSU.

The epistemic motivation relates to an individual's or group's understanding and knowledge of a phenomenon; conspiracy theories can allow individuals to preserve a sense of understanding in the face of uncertainty and contradiction. These beliefs are noted to become stronger when events are widespread and/or significant,^12^ and when simplistic, mundane explanations are perceived as unsatisfactory.^13^ Conspiracy beliefs can foster a sense of cognitive closure when the situation lacks a clear, consistent and understandable official message.^14^

When individuals feel anxious, threatened and powerless in the face of danger, they may gravitate towards conspiracy theories to achieve a sense of comfort.^11,15,16^ These are viewed as existential motivations.^11^ Such powerlessness can be exaggerated by a perception of alienation from decision makers and a breakdown in containment and social order.^7,15^

Douglas et al^11^ note that social motivations also contribute to the formation of conspiracy beliefs. Groups that have experienced persecution, for example victims of police harassment^17^ or racial discrimination,^18^ are more likely to perceive dominant groups as conspiring against them. Research has shown that members of low-status groups are more likely to endorse conspiracy theories than those of higher status.^15,19^ In-group attachments can strengthen in the face of group threat, and ‘collective narcissism’ (an emotional investment in an unrealistic belief about the in-group's greatness)^20^ can develop, particularly when underprivileged, undervalued and under threat.^19^ This may function to protect the in-group by forming a shared ‘us versus them’ narrative.^21^ Similarly, individual narcissism is understood to emerge as a defence in response to perceived powerlessness; a conspiracy theory is powerful as it ascribes ‘special knowledge’ to the believer, imbuing a safe sense of superiority.^22^

Such motivations and psychosocial characteristics are relevant and, in some circumstances, exaggerated in the MSU population. For example, research has linked subclinical delusional thinking^23^ and schizotypy^24,25^ to conspiracy thinking. Individuals diagnosed with paranoid personality disorder demonstrate similar conspiratorial thinking.^26,27^ Cognitive/affective mechanisms at play in such samples are also relevant to those at the distressing/impairing end of the psychosis continuum, i.e. those diagnosed with schizophrenia.^28^ For example, the omission of true cognitive information^29^ could precipitate a jumping to conclusions (JTC) bias that is associated with the rapid appraisal of ambiguous or anomalous stimuli to form a conclusion without a sound evaluation of evidence.^30^ Such a bias is evident in subclinical^31^ and clinical populations.^32^ Moulding et al^33^ have identified that holders of conspiracy beliefs are more likely to view the world as threatening. Such schematic views of the world as dangerous^34^ can underpin the process whereby delusional beliefs – in an attempt to secure cognitive closure – form from misappraisals of anomalous stimuli.^30^ Of note, a high proportion of our in-patient population hold – or have held – delusional beliefs.

Disproportionately, MSU in-patients have been exposed to early life danger^35^ and hold negative schematic beliefs about self, others and the world.^36^ Psychotic delusions, conspiracy theory beliefs and self-protective distortions have a propensity to surface when conditions are dangerous and uncertain.^15,16^

## The impact of the pandemic within the clinic

Meme theory can help to explain how such ideas spread, particularly in contained environments. Dawkins^37^ considered memes to be cultural phenomena that pass from one mind to another, and survive (or die) through a process analogous to genetic selection. Goertzel^38^ noted ‘conspiracy theorizing [*sic*] is a rhetorical meme that transforms scientific controversies into human dramas with villains who can be exposed’.

In the general population, COVID-19 conspiracy theory memes (e.g. 5G phone masts, man-made virus) have gone viral, with some harmful and persistent consequences. More broadly, memes that run as counter-narratives to the government's explanations and advice affect some people, who may then spread their ideas to others. This may lead to a failure to act according to government guidelines and in the best interest of public health.^6^

We have observed conspiracy theory memes to develop in two distinct ways within the MSU. First, ‘organic memes’ have developed on one ward. These have taken the form of a belief that the pandemic is orchestrated by the hospital staff to restrict leave and delay discharge. Such a belief is likely to have formed with no outside influence and is perhaps good evidence that humans will seek conspiracy theory explanations in isolation to allay epistemic, existential and social concerns.^11,19^ It is our view that, despite several men endorsing this meme to a greater or lesser degree, it will likely wither and fail to spread owing to its fallibility in the face of simple counter-evidence and the physical health restrictions that prohibit mixing of wards (this meme is unlikely to be shared by staff members).

The type of second conspiracy meme is more problematic and harder to contain. These are externally generated conspiracy theories. Such memes may find traction among the internal population by direct or indirect conversations, through telephone contact, media consumption and where there are exchanges of perspectives. It is not possible – or ethical – to stop the introduction of conspiracy theory memes via telephone contact with relatives. However, staff members may be prone to conspiracy beliefs because of their own sense of powerlessness, threat and existential anxiety. As staff members move around the MSU, there is a risk of them spreading conspiracy beliefs to others. Additionally, misinformation might be introduced into the hospital. This new discrepant information may destabilise an already vulnerable in-patient population and prime conspiratorial thinking.

## The impact of a COVID-19 conspiracy theory meme outbreak

Healthy secure wards are able to maintain a negotiated homeostasis, whereby clear boundaries and good clinical practice maintain order, safety and containment, while also promoting mental health rehabilitation. Conspiracy theory memes present a threat to this architecture. A possible consequence is a breakdown in trust and cohesion, which would undermine physical and psychological safety, and challenge measures to contain the virus.^6^

Uncertainty and unpredictable danger can be precipitants of anxious threat states. Changes in routine or the introduction of new conditions can trigger a loss of perceived environmental control and subsequent attempts to regain safety. As such, periods of stress and threat require the use of automatic self-protective behaviours and implicit information processing strategies.^29^ Harmful conspiracy theories or hypotheses can increase uncertainty and decrease trust in authority figures. For many men in forensic in-patient settings, violence or self-harming behaviour has been – or is – an adaptive part of their self-protective behavioural repertoire. When in conditions of threat, such behavioural expressions might manifest to gain control, discharge arousal, communicate distress or elicit care.

Similarly, splitting is a possibility, with competing memes generating an ‘us versus them’ dynamic. As described previously, this is an evidenced component of conspiracy theory motivation, and staff members can become targeted if inequality is perceived (e.g. locked down versus transient, exposed versus PPE). Systemically, these processes can heighten the sense of danger for other residents and group anxiety can escalate. Of course, staff members are not immune to such effects and negative consequences are possible (e.g. burnout, increased punitiveness).

## The response

Memes are hypothesised to spread in a manner analogous to a virus.^37,38^ Hence, we propose that a fast, stringent and proactive strategy is required to curb the sharing of unhelpful and false memes. We suggest that the response to ‘prevent’ and ‘treat’ conspiracy theories can be pitched according to the public health approach to diseases: primary, secondary and tertiary prevention.

Importantly, some degree of uncertainty is unavoidable owing to a global lack of clarity regarding COVID-19. It has to be acknowledged that there are few unambiguously *true* known facts about the virus. We do not advocate the suppression of questioning or critical challenge of official narratives. A host of different memes, differing in strength, transmissibility and potential harmfulness, will spread among staff and patients. We recommend that professionals demonstrate clinical judgement to determine if and when intervention is required and listen to alternative perspectives, discussing them in context.

### Primary prevention

Primary prevention aims to prevent disease or injury before it occurs. To prevent the development of conspiracy theories within an MSU, we recommend addressing the conditions that lead to such thinking.

We consider the first line of response to be education. Conspiracy theory memes are hypothesised to breed from indecision and uncertainty; gaps in knowledge allow room for a counter-narrative to develop to fulfil a need for cognitive closure^14^ and a perception of control.^11,15,16^ We view the regular and consistent dissemination of clear and transparent information about the pandemic, the ‘outer world’ situation and MSU policy to be essential to maximise patients’ knowledge. Information can be adapted to account for complex communication needs, and care plans developed accordingly. Ideally, patients who are vulnerable to being affected by conspiracy beliefs should be identified and bespoke assessments and management plans completed.

The staff group are not immune from conspiratorial thinking. Helping staff members to feel informed requires the consistent dissemination of information in a manner that is accessible to all. Changes in practice should be quickly communicated. Information should be transparent, with an open forum approach to address queries and signpost to relevant resources. In addition, an honest acknowledgement of challenges that individuals and teams will face is necessary to ensure preparedness. To prevent splitting and/or ‘suffering in silence’, regular reflective practice, peer group supervision and *ad hoc* ‘check ins’ can give space for the processing of anxiety and an opportunity to work through uncertainties and questions. Greater use of virtual connectivity has allowed sick or shielded colleagues to sustain communication with core teams, thus maintaining a collective ‘togetherness’.

Research has suggested that it is important that education provided for staff and patients is presented in an ‘even-handed’ manner (i.e. do not dismiss counter-narratives offhand) to prevent the perception of indoctrination or bullying.^39,40^ Failure to do this successfully could lead to the educator being absorbed into the conspiracy belief.^41^ Information sharing might take the form of standardised and accessible information boards, regular ward ‘community meetings’ and individual conversations with patients and staff to ensure that they feel informed about events.

In our NHS trust, daily staff briefings have been provided by the chief executive officer. There are daily meetings held by senior management within the MSU to strategise, coordinate a unified response and ensure that information is shared – and then cascaded – evenly throughout the site. In addition, members of different clinical disciplines have adapted their roles. For example, individual psychologists have ‘cohorted’ to provide intensive support for single wards, occupational therapists have provided opportunities for activity and release from the claustrophobic ward spaces and the psychiatric team have employed a ‘shadow rota’ to ensure that sickness does not reduce the provision of emergency care. Collectively, these additions and adaptations to practice can be understood as ‘inoculation’ of the community.^42^ Many of these organisational strategies are likely to be in place to serve other, important needs. However, it is our view that such good practice is also relevant to the aims of this paper.

### Secondary prevention

The aim of secondary prevention is to reduce the impact of a disease or injury that has already occurred. We recommend that conspiracy theories already in circulation should be identified at the earliest possible point and the conveyance slowed. The ultimate aim is to challenge unhelpful or disruptive memes that threaten to break down cohesion in the MSU community. Strategies need to prevent re-emergence and reconnect those affected to a less detached position. However, if this is not possible, the focus shifts to containment and reduction of the spread to others.

The infection control response to COVID-19 (i.e. ‘lockdown’ of wards) will inadvertently prevent the cross-contamination of conspiracy theory memes across the MSU site. However, conspiracy theories can infiltrate the community via telephone calls, media and/or staff acting as vectors. It is clearly counterintuitive, unethical and disproportionate to restrict or monitor private phone calls. Secondary prevention should therefore be targeted at the management of memes that are conspiratorial in nature or undermining of national or local COVID-19 policies.

We recommend that changes in anxiety, mood and behaviour associated with conspiracy thinking – or exposure to such ideas – should be observed as part of the usual monitoring of mental state. In the MSU, all patients are regularly reviewed by the nursing staff and forensic psychiatrists, who examine their mental states and the extent of psychopathology. Patients can be given space to explore their thoughts and feelings about such theories; the clinician can then establish whether intervention is required. A ‘COVID-19 formulation-led’ approach to addressing concerns as they arise is recommended.

When discussing conspiracy theories – or related memes – information should be presented in a consistent, clear and accessible manner so that further doubt, ambiguity or reinforcement of the conspiracy does not result.^41^ The patient will require adequate knowledge to close the ‘uncertainty gap’. This work may also be achieved through group or individual therapy sessions. In our MSU, we have found that acceptance and commitment therapy (ACT) principles have been beneficial, particularly as these can address issues relating to control and uncertainty.^43^ In addition, mindfulness practice can help to calm heightened arousal states, release troubling thoughts and teach self-awareness.^44^ As uncertainty is largely inescapable, such therapeutic approaches are preferable to the suppression of all but the most harmful memes. Cognitive remediation strategies can improve reasoning ability^45^ and various non-verbal therapies can help to up- or down-regulate arousal.

Considering the ward as a whole, the maintenance of a ‘safe’ and ‘cohesive’ environment is vital to prevent the harmful effects of conspiracy theory memes. The basis for this is already provided via the implementation of the ‘Safewards’ approach^46^ and positive behavioural support planning.^47^ Indeed, an approach not dissimilar to trauma-informed care could be adopted: ‘pandemic-informed care’ would incorporate the necessary physical health precautions, while also proactively identifying and addressing the emergence of conspiracy theory memes and promoting a clinical awareness of the vulnerabilities of patients who are prone to engage in conspiracy thinking. Pandemic-informed care would also include the provision of staff support and reflective practice.

Some patients who have been exposed to conspiracy theories may become paranoid, anxious or distressed in response to this exposure. If there is a resultant significant decline in symptoms and functioning in which the expression of delusional ideas and other psychotic features is identified, there are a range of pharmacological, psychological and risk-management techniques that may need to be considered.

### Tertiary prevention

Tertiary prevention is a strategy to reduce the impact of an ongoing illness or injury that has lasting effects. By definition, many individuals who are resident in an MSU experience complex psychopathology and are vulnerable to anxiety, paranoia and conspiratorial beliefs. Clinicians involved in their care are well advised to consider the impact of ‘lockdown’, uncertainty and competing narratives, and the destabilising effect that each might have.

A multidisciplinary approach is required to incorporate such formulations into care planning and intervention, as has been undertaken across our MSU. In acutely psychotic patients – and those susceptible to relapse – there is a risk that COVID-19-related fears could become enmeshed with pre-existing delusional belief systems. In a patient who becomes absolutely engrossed by conspiracy beliefs to the point that it manifests as a delusion and/or other features of a psychosis and significantly affects their function, an individual, tailored approach must be adopted. The priority would be the containment of severe pathological symptoms, with consideration given to pharmacological and risk management interventions. In addition, the reinforcement of a consistent and safe environment is necessary to allow the individual to feel secure and grounded. Access to regular, trusted and familiar nursing staff is likely to be important. Arousal-regulating therapy might also be considered. Deterioration in mental state may prompt a review of the patient's current setting. A decision may need to be taken as to whether an acute ward may be more appropriate or higher levels of observations needed. In each case, the acute symptomatology needs to be addressed and, in time, once stable, measures taken to challenge the conspiracy thinking via psychological intervention.

As regards measures introduced to support staff, it is recommended that these are maintained beyond the acute phase of the response. Conspiracy theory memes might retract while the various levels of intervention are in place. However, if support is withdrawn too quickly, a breakdown in communication, increased isolation and potential feelings of abandonment (that accompany burnout) might prompt disharmony and a failure to adhere to the previously outlined necessary actions. This may lead to a second wave of conspiracy beliefs emerging.

## Conclusions

These are extraordinary times in society and clinical practice; there is a heavy emphasis on how to identify and manage the physical health manifestations of COVID-19 among the general population, existing patients and the workforce. However, COVID-19-associated conspiracy theory memes also present a societal challenge, which is perhaps exaggerated in a forensic in-patient setting. There is nuance as to what memes should be challenged and the degree to which challenge is made. This is a clinical decision on a case-by-case basis. However, a failure to intervene in an appropriate, effective and ethical manner when memes are potentially harmful could precipitate a breakdown in therapeutic relationships, ward cohesion and the successful implementation of physical health procedures. The consequences of such breakdown relate to mental health deterioration, increased risk behaviours and the failure to curtail the spread of COVID-19. Below, we make suggestions that are consistent with the conspiracy theory literature, which may be helpful to manage the development and spread of conspiracy theory memes (we also consider this guidance to be applicable to other custodial settings, such as prisons):

- provide clear, consistent and up-to-date information to patients and staff
- clearly explain the rationale for change (e.g. new practices/restrictions)
- empower staff and patients to make informed decisions in relation to care
- develop bespoke multidisciplinary COVID-19 formulations for each patient
- regularly review patients with reference to their experience of COVID-19
- ensure that all clinical environments are ‘safe spaces’ and that interactions are therapeutically informed (pandemic-informed wards)
- provide psychological intervention to address uncertainty, change and anxiety, and consider the use of cognitive remediation strategies to enhance reasoning
- be prepared to utilise short- and long-term pharmacological and risk-management strategies as required if mental state deteriorates significantly
- maintain team cohesion through regular reflective practice, peer supervision and *ad hoc* individual ‘check ins’
- provide appropriate challenge to conspiracy theory memes, with an awareness of the theory outlined in this paper.

## About the authors

**Reena Panchal** is a Specialty Trainee Year 5 (ST5) in forensic psychiatry in the Department of Psychiatry, Reaside Clinic, Birmingham and Solihull Mental Health Foundation Trust, UK. **Alexander Jack** is a senior forensic psychologist in the Department of Psychology, Reaside Clinic, Birmingham and Solihull Mental Health Foundation Trust, UK.
